# Supplementary figures and images for: Temporal Trend of the SARS-CoV-2 Omicron Variant and RSV in the Nasal Cavity and Accuracy of the Newly Developed Antigen-Detecting Rapid Diagnostic Test
Source: Diagnostics (Basel). 2024 Jan 4;14(1):119. doi: 10.3390/diagnostics14010119 (PMC10802845; doi:10.3390/diagnostics14010119)

Supplementary Figure S1. Method of RapidTesta RSV and SARS CoV-2 using RapidTesta Reader

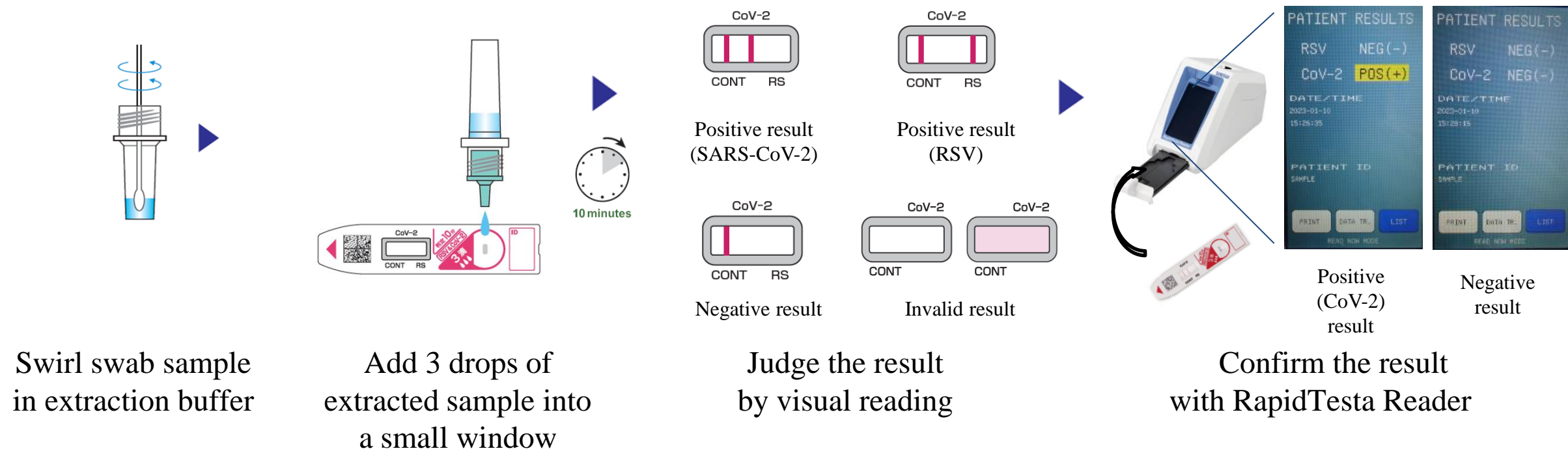

Supplement: Supplementary file 1 [file diagnostics-14-00119-s001.zip › diagnostics-2771826-supplementary.pdf]
